# TIM-3 teams up with PD-1 in cancer immunotherapy: mechanisms and perspectives

**DOI:** 10.1186/s43556-025-00267-6

**Published:** 2025-05-07

**Authors:** Zhuohong Yan, Chunmao Wang, Jinghong Wu, Jinghui Wang, Teng Ma

**Affiliations:** 1 Department of Cancer Research Center, Beijing Chest Hospital, Capital Medical University, Beijing Tuberculosis and Thoracic Tumor Research Institute, Beijing, 101149 China; 2 Department of Thoracic Surgery, Beijing Chest Hospital, Capital Medical University, Beijing, 101149 China

**Keywords:** Immune checkpoint inhibitors, TIM-3, PD-1/PD-L1, Immunotherapy, Combined therapy, Clinical trials

## Abstract

Immunotherapy using immune checkpoint inhibitors (ICIs) has become a prominent strategy for cancer treatment over the past ten years. However, the efficacy of ICIs remains limited, with certain cancers exhibiting resistance to these therapeutic approaches. Consequently, several immune checkpoint proteins are presently being thoroughly screened and assessed in both preclinical and clinical studies. Among these candidates, T cell immunoglobulin and mucin-domain containing-3 (TIM-3) is considered a promising target. TIM-3 exhibits multiple immunosuppressive effects on various types of immune cells. Given its differential expression levels at distinct stages of T cell dysfunction in the tumor microenvironment (TME), TIM-3, along with programmed cell death protein 1 (PD-1), serves as indicators of T cell exhaustion. Moreover, it is crucial to carefully evaluate the impact of TIM-3 and PD-1 expression in cancer cells on the efficacy of immunotherapy. To increase the effectiveness of anti-TIM-3 and anti-PD-1 therapies, it is proposed to combine the inhibition of TIM-3, PD-1, and programmed death-ligand 1 (PD-L1). The efficacy of TIM-3 inhibition in conjunction with PD-1/PD-L1 inhibitors is being evaluated in a number of ongoing clinical trials for patients with various cancers. This study systematically investigates the fundamental biology of TIM-3 and PD-1, as well as the detailed mechanisms through which TIM-3 and PD-1/PD-L1 axis contribute to cancer immune evasion. Additionally, this article provides a thorough analysis of ongoing clinical trials evaluating the synergistic effects of combining PD-1/PD-L1 and TIM-3 inhibitors in anti-cancer treatment, along with an overview of the current status of TIM-3 and PD-1 antibodies.

## Introduction

Cancer treatment has witnessed the rise of a crucial approach-immunotherapy, making significant progress in the field. The identification and targeting of immune checkpoint molecules address the limitations of traditional treatments. Both stimulatory and inhibitory molecules are essential for maintaining the delicate immune system’s balance [1]. Immune checkpoint inhibitors (ICIs) have successfully transferred from experimental research to clinical application, demonstrating promising efficacy in treating specific cancers. Ipilimumab, the first ICI that targets cytotoxic T lymphocyte antigen 4 (CTLA4), was authorized by the Food and Drug Administration (FDA) in 2011. FDA subsequently approved inhibitors targeting programmed cell death protein-1 (PD-1), programmed death-ligand 1 (PD-L1), and lymphocyte activation gene 3 (LAG-3). However, despite these significant advancements, the clinical response rate to ICIs remains suboptimal.

Inhibitory immune checkpoint molecules, including PD-1, CTLA4, LAG-3, T cell immunoglobulin and mucin-domain containing-3 (TIM-3), and T cell immunoreceptor with immunoglobulin and immunoreceptor tyrosine-based inhibitory motif (ITIM) domain (TIGIT), primarily exert immunosuppression function. In contrast, stimulatory immune checkpoint molecules such as CD28, CD137, and OX40 play pivotal roles in the activation of immune response. The unrestricted T cell activation is suppressed by the up-regulation of inhibitory checkpoint molecules. ICIs are therefore designed to block inhibitory receptors, and reactivated T cells. The presence of additional immune checkpoint molecules significantly contributes to the currently suboptimal response rates to immunotherapy, thereby highlighting the critical need for new therapeutic approaches and combination strategies [2–4].

TIM-3 represents a novel and highly promising inhibitory checkpoint molecule in the field of cancer immunotherapy [5–8]. In diverse cancer, TIM-3 expression is upregulated as a result of PD-1 inhibition. A strong correlation has been established between TIM-3 overexpression and resistance to PD-1 therapy [3, 9–12]. TIM-3 inhibits T and natural killer (NK) cell responses by interacting with its ligands, thereby facilitating tumor cells’ evasion of immune surveillance [13]. Research has shown that inhibiting TIM-3 can effectively restore the activity of T and NK cells [14–16]. Additionally, it has been established that tumor cells express TIM-3 [17–19], playing a pivotal role in modulating their malignant behaviors [18]. It is worth noting that most leukemic stem cells in acute myeloid leukemia (AML) express TIM-3 [20–23]. Blocking TIM-3 exerts dual effects on both immune cells and tumor cells [19]. The investigation of TIM-3 monoclonal antibodies (mAbs), including MBG453, TSR-022, and Sym023, is now underway in early-stage clinical trials [6].

TIM-3 and PD-1 are expressed at distinct phases of the differentiation process of exhausted T cells in cancer. They have a major impact on the efficacy of immunotherapy and are essential in controlling T cell exhaustion [24]. Patients diagnosed with advanced solid tumors treated with combination therapy comprising spartanzumab (anti-PD-1 mAb) and sabezumab (anti-TIM-3 mAb) have shown a decent safety profile and encouraging anti-tumor benefits in phase I/II clinical studies [2, 25]. It is anticipated that TIM-3 blocking combined with anti-PD-1/PD-L1 therapy will significantly improve therapeutic efficacy and overcome drug resistance [26].

This review comprehensively overviews the biological characteristics and function of TIM-3 and PD-1 in cancer immunology, elucidates the underlying mechanisms, examines the combined effects of co-inhibiting PD-1/PD-L1 and TIM-3, and evaluates ongoing clinical studies involving this combination treatment.

## TIM-3 and PD-1: basic biology

### TIM-3

First identified in 2002, TIM-3 exists in CD4^+^ T helper 1 (Th1) and CD8^+^ cytotoxic T lymphocytes (CTLs) that produces interferon-gamma (IFNγ) [27]. Subsequently, its expression in various other immune cell types has been documented, including activated NK cell [28], Th17 cell [29], gamma delta (γδ) T cell [30–33], regulatory T cell (Treg) [34], macrophages [27], dendritic cells (DCs) [35], and mast cell [36].

TIM-3 refers to the TIM family, which comprises three members in human: TIM-1, TIM-3, and TIM-4, and they are each encoded by the genes *HAVCR1*, *HAVCR2*, and *TIMD4*. TIM-1 through TIM-8 are the eight distinct members of the mouse TIM family [37]. Among these, TIM-3 holds a distinct position due to its pivotal role in modulating immunological responses, especially in the setting of cancer [38, 39].

#### TIM-3 structure and its ligands

**Fig. 1:**
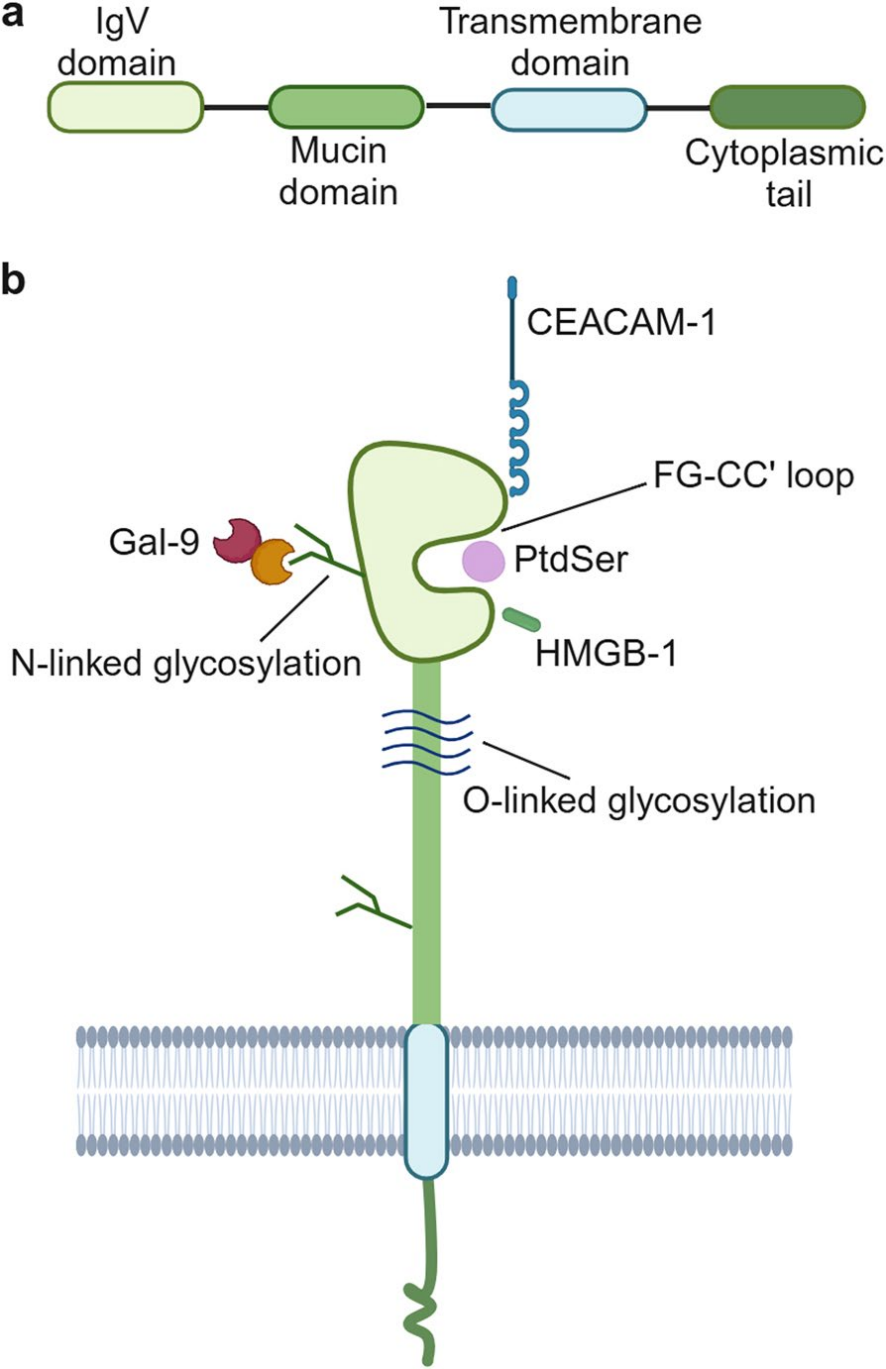
Schematic diagram of the TIM-3 molecular structure. **a** TIM-3 comprises four distinct domains: the IgV domain, Mucin domain, Transmembrane region, and Cytoplasmic region. **b** TIM-3 on immune cells interacts with various ligands, including Gal-9, CEACAM-1, PtdSer, and HMGB-1. Gal9: galectin-9; CEACAM-1: carcinoembryonic antigen-related cell adhesion molecule 1; HMGB-1: high-mobility group box 1 protein; PtdSer: phosphatidylserine

The TIM-3 gene is situated at locus 5p33.3 in the human genome [40] and chromosome 11B1.1 in the mouse genome. The human TIM-3 protein consists of 301 amino acid residues, while its murine counterpart comprises 281 amino acid residues. There is a 63% sequence homology between the two species [13]. One immunoglobulin variable (IgV) domain, a mucin stalk domain, a single transmembrane domain, and a cytoplasmic tail domain constitute the usual structure of TIM-3, a type I transmembrane protein (Fig. 1) [6, 41, 42]. The N-linked glycosylation sites and FG-CC’ loop are features of IgV domain. The mucin stalk domain region contains both N-linked and O-linked glycosylation sites.

Four TIM-3 ligands interact with TIM-3 IgV domain (Fig. 1 and Table 1): (1) galectin-9 (Gal-9) [43], which is considered the most relevant, induces apoptosis of Th 1 cells through intracellular calcium; (2) phosphatidylserine (PtdSer) [44], a major membrane phospholipid often found on the cell membrane’s inner leaflet but becomes exposed to outside leaf during apoptosis, is necessary for the apoptotic particle clearance and facilitates antigen cross-presentation [45]; (3) high-mobility group box 1 protein (HMGB-1) [35] triggers innate immune responses through DCs by binding to nucleic acids released by dying tumor cells. By competing with nucleic acids for binding to HMGB-1, TIM-3 reduces the amount of nucleic acids that are delivered to the nucleosome and thus inhibits the innate immune response that tumor-associated nucleic acids trigger [35]; and (4) carcinoembryonic antigen-related cell adhesion molecule 1 (CEACAM-1) [46], which serves as a self-ligand on T cells, exerts negative modulation on T cell responses [37, 41].

**Table 1:** Basic characteristics of TIM-3 and PD-1 receptors [42, 47]

Checkpoint	Alternative name	Signaling motif	Ligand	Associated phosphatase
TIM-3	CD366, HAVCR2	Tyrosine Y265 and Y272 in human; Y256 and Y263 in mice	Gal-9, PtdSer, HMGB-1, CEACAM-1	—
PD-1	CD279, PDCD1	ITIM, ITSM	PD-L1 (CD274; B7-H1), PD-L2 (CD273; B7-DC)	SHP-2

TIM-3’s ligands recognize distinct areas of its extracellular IgV domain. The FG-CC' loops specifically serve as binding sites for PtdSer, CEACAM-1, and HMGB-1 [35, 48]. On the other side of TIM-3's FG-CC’ face, the binding site for Gal-9 is different and is expected to be made up of N-linked glycans.

#### Signal transduction of TIM-3

In the battle against cancer, the effective activation of the immune system is crucial, particularly the potent effector function of cytotoxic CD8^+^ T cells. These T cells initiate an immunological response through two distinct signaling pathways: the T cell receptor (TCR) signaling pathway and the costimulatory signaling pathway. The activation of CD8^+^ T cells involves the interaction between the major histocompatibility complex (MHC)-antigen peptide complex presented on antigen-presenting cells (APCs) and the T cell receptor (TCR). Additionally, B7 ligands (B7.1/CD80 and B7.2/CD86) expressed on APCs engage with the primary co-stimulatory molecule CD28 on T cells, which is essential for the initiation of T cell response [49]. This activation results in T cell expansion, improved functional capabilities, and the establishment of long-term memory [50].

TIM-3, a coinhibitory molecule, interacting with its ligands mediates T cell inhibition. In contrast to other immune receptors, such as PD-1 [42, 51] and TIGIT [5], TIM-3 lacks the typical inhibitory immunoreceptor tyrosine-based inhibition motifs (ITIMs) or immunoreceptor tyrosine-based switch signaling motifs (ITSMs). Notably, the cytoplasmic tail of TIM-3 has five tyrosine residues. Although the exact intracellular signaling mechanisms of TIM-3 remain incompletely elucidated, it has been established that in humans, the downstream signaling pathway hinges on the Y265 and Y272 tyrosine residues. These two tyrosine residues are surrounded by conserved peptide sequences in both humans and mice, serving as Src homology 2 (SH2) domains binding sites. Several kinases harboring SH2 domains have been reported to bind to these sequences, including Src kinase Fyn and Lck, interleukin-induced T cell kinase (Itk), and the phosphatidylinositol 3-kinase (PI3K) p85 adaptor protein [13, 40, 52]. A functional connection between the TCR pathway and TIM-3 is suggested by the fact that many of these molecules are essential components within the TCR signaling pathway [13].

The tyrosine residues Y265 and Y272 (Y256 and Y263 in mice) of TIM-3 are phosphorylated by Itk and Src family kinases upon their interaction with the ligands Gal-9 or CEACAM-1. This results in the detachment of human leukocyte antigen B (HLA-B)-associated transcript 3 (Bat3) from the TIM-3 protein complex. Following this release, TIM-3 binds to Fyn, a Src family kinase that shares the same binding site as Bat3 [42, 53]. This interaction promotes Fyn-mediated recruitment and phosphorylation of phosphoprotein associated with glycosphingolipid microdomains 1 (PAG1), a phosphorylation-sensitive adaptor protein that recruits C-terminal Src kinase (Csk). Csk subsequently phosphorylates Lck’s C-terminal tyrosine residues, thereby suppressing Lck kinase activity and dampen TCR signaling [6, 40, 42, 54, 55]. Additionally, by attenuates B7/CD28 co-stimulation signals, this regulatory cascade significantly inhibits T cell activation and cytokine production.

**Fig. 2:**
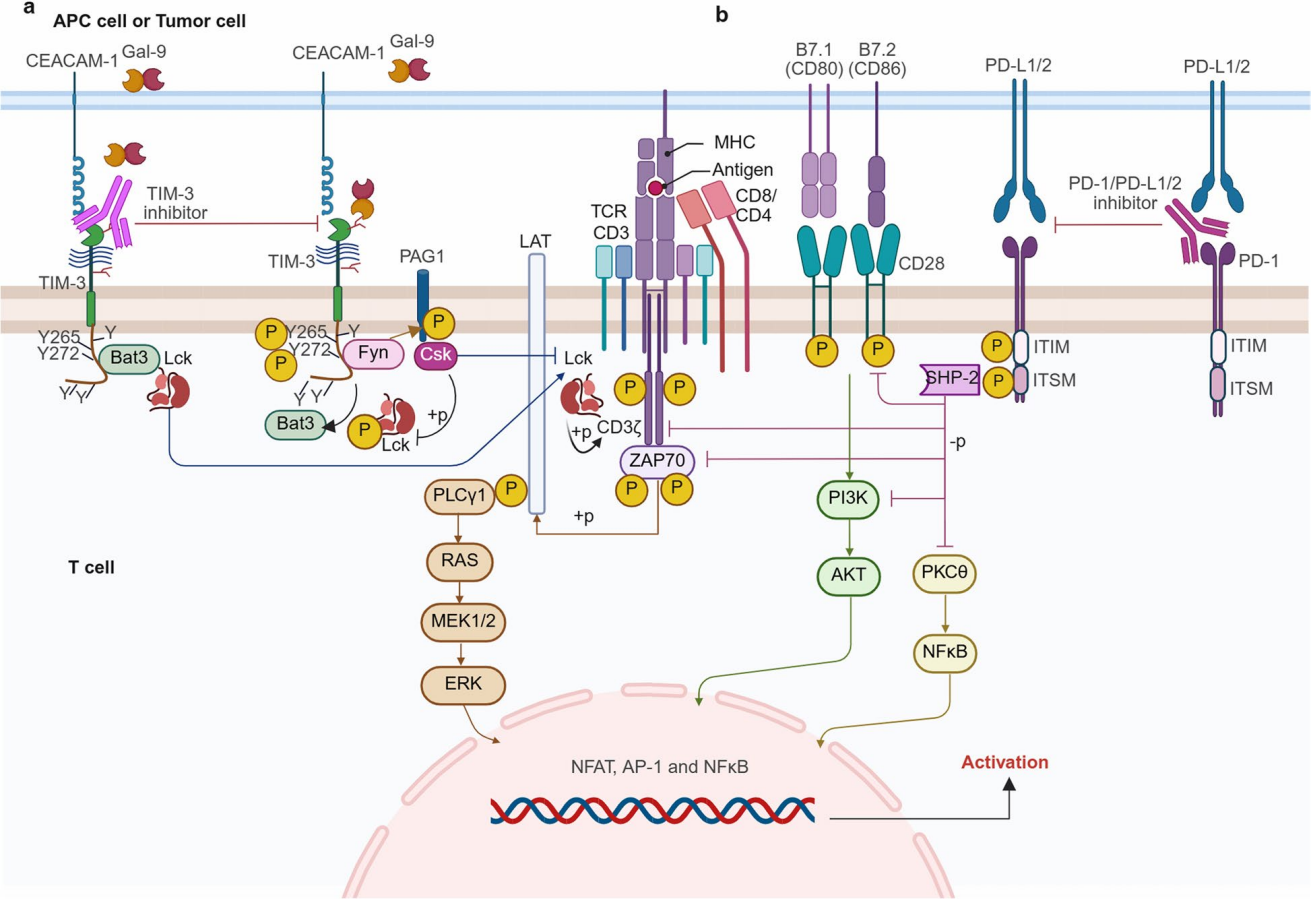
TIM-3 and PD-1 pathways and their inhibitors. **a** TIM-3 pathway and its inhibitor. T cell activation requires two distinct signals. Upon the antigen-presenting cell (APC) presenting the MHC-peptide complex to the TCR, a primary activation signal is generated. Subsequently, lymphocyte-specific protein tyrosine kinase (Lck) phosphorylates TCR’s CD3, activating the 70-kDa zeta-associated protein kinase (ZAP70). Phosphorylated linker for activation of T cells (LAT) then binds and activates phospholipase Cγ1 (PLCγ1), a signaling effector crucial for the generation of second messengers. The B7 ligand on APC interacts with CD28 on T cells to provide a costimulatory signal. In response to both primary and costimulatory signals, essential transcription factors are activated and translocated to nucleus, facilitating target gene transcription. TIM-3 interacts with its ligands mediate immune suppression. When TIM-3 on T cells interacts its ligand Gal-9 or CEACAM-1 on APCs, intramembrane residues Y265 and Y272 of TIM-3 are phosphorylated by Itk, resulting in the release of human leukocyte antigen B (HLA-B)-associated transcript 3 (Bat3) from TIM-3 and subsequent recruitment of Fyn and C-terminal c-Src kinase (Csk). Csk subsequently phosphorylates Lck’s C-terminal tyrosine residues, thereby suppressing Lck kinase activity and dampen TCR and B7/CD28 signaling. TIM-3 inhibitor obstructs the interaction between TIM-3 and its ligands. This prevents the phosphorylation of TIM-3’s intramembrane motif. In the presence of TIM-3 inhibitors or in the absence of a ligand, TIM-3’s Y265 and Y272 interact with Bat3, recruiting the catalytically active form of Lck, which phosphorylates TCR and CD28 to restore their activation. **b** PD-1 pathway and its inhibitor. PD-L1/2 on APCs or tumor cells interact with PD-1 on T cells, inhibiting T cell function. Upon PD-1 activation, the tyrosine residue in PD-1’s ITSM became phosphorylated, leading to recruitment of Src homology 2 domain-containing protein tyrosine phosphatase 2 (SHP-2). SHP-2 subsequently dephosphorylates of crucial molecules involved in CD3ε, ZAP70, and phosphatidylinositol 3-kinase (PI3K) and protein kinase C-theta (PKCθ), downregulating TCR, CD28, PI3K-AKT, RAS-MEK-ERK, and PKCθ-NFκB pathways. PD-1/PD-L1/2 inhibitors prevent these cascade reactions, thereby enhancing T cell activation. Specifically, PD-1/PD-L1/2 inhibitors prevent the intramembrane motif of PD-1 from being phosphorylated by Lck, thus blocking the recruitment of SHP-2 and preventing the dephosphorylation of downstream pathways. NFAT = nuclear factor of activated T cells; AP-1 = activator protein 1; NFκB = nuclear factor-kappa B

In the absence of a ligand or in the presence of TIM-3 inhibitors, TIM-3 residues Y265 and Y272 bind with Bat3 [40, 53]. This interaction results in the recruitment of the catalytically active Lck (Fig. 2a) [6, 42]. Afterwards, clustered Lck phosphorylates the CD3 domain of the TCR, which in turn recruits and activates 70-kDa zeta-associated protein kinase (ZAP70) [56]. Next, ZAP70 phosphorylates linker for activation of T cells (LAT) [57, 58], leading to the recruitment and activation of phospholipase Cγ1 (PLCγ1). PLCγ1 is a critical signaling effector that contributes to the generation of second messengers, which in turn trigger the nuclear factor of activated T cells (NFAT), MEK/ERK, and nuclear factor kappa B (NFκB) pathways, thereby promoting T cell activity [42]. TIM-3 facilitates T cell activation in this situation [52]. T cell activity is reportedly inhibited when TIM-3 co-localizes at the immunological synapse with CD45 and CD148 [59, 60].

Recently, studies have demonstrated that the interaction between PtdSer and TIM-3 modulates the production of interleukin-2 (IL-2) and regulates the activity of the NFκB pathway in TCR-activated Jurkat cells [61]. The engagement of CEACAM-1 with TIM-3 suppresses NFκB signaling in macrophages [62]. The binding of HMGB-1 to TIM-3 inhibits the activation of CD4^+^ T cells [63] and DC cells [35] by suppressing NFκB signaling. Additionally, the interaction of PtdSer with TIM-3 attenuates PI3K/AKT/mTORC1 signaling pathways, thereby diminishing cytotoxin capability of NK cells [64].

#### The physiological functions and roles of TIM-3 in immune cells

TIM-3 plays a significant role in modulating both innate and adaptive immunological responses by acting as a negative regulator of immune cells. It promotes immunological tolerance by inducing T cells apoptosis or impeding the activation of innate immune cells [65, 66]. Dysregulation or malfunction of TIM-3 has been connected to the exacerbation of autoimmune diseases in various preclinical models [5, 13], including inflammatory bowel disease [67], experimental autoimmune encephalomyelitis (EAE) [27, 68], and diabetes [69]. Clinical research has demonstrated that the expression of TIM-3 on T cells is markedly reduced in patients diagnosed with ulcerative colitis [70], multiple sclerosis (MS) [71, 72], and psoriasis [73] than in healthy controls.

TIM-3’s unique binding patterns with its ligands in different cell types result in several significant biological outcomes (Table 2). Specifically, TIM-3 and Gal-9 interaction causes Th1 cells and CD8^+^ CTLs to undergo apoptosis [24, 76]. Furthermore, TIM-3’s interaction with either Gal-9 or CEACAM-1 enhances Treg cell activity while simultaneously weakening CD8^+^ T and Th1 cell function, leading to T cell exhaustion. It has been shown in recent research that TIM-3 suppress γδ T cell’s activity in cancers [31, 33].

**Table 2:** The interaction and function of TIM-3 and its ligands in immune cells

Receptor/Ligands	Cell types	Biological function of Receptor-Ligand interaction
TIM-3/Gal-9	Th1	Negatively regulates IFNγ secretion [43]; Suppresses Th1 effector function [74]; Gal-9 controls differentiated TIM-3^+^ Th1 cells in vivo [75]
	Treg	Enhances suppressive capacity of Treg cells [65, 74]
	CD8^+^ T	Induces CD8^+^ CTLs apoptosis [24, 76]; Mediate CD8^+^ T cell exhaustion caused by MDSCs [77]
	NK	Induces NK cell exhaustion [78, 79]
	Macrophages	Regulate pro-tumor M2-like polarization [11, 80–82]
TIM-3/PtdSer	CD8^+^ T	TIM-3^+^APCs interacts with PtdSer on CD8^+^ TILs and tumor antigen-specific CD8^+^ T cells, limiting antitumor immunity through T cell trogocytosis [83]
	DCs	Aids in TIM-3^+^DCs’ function in improving antigen cross-presentation and promoting apoptotic body elimination [44]
TIM-3/HMGB1	DCs	Suppresses the innate immunological responses mediated by nucleic acids [35]; Suppresses HMGB1-dependent DNA sensing in intra-tumoral dendritic cells through the cGAS-STING pathway [84]
TIM-3/CEACAM1	CD8^+^ T	Mediate tolerance and T cell exhaustion [46, 85, 86]

*CTLs* Cytotoxic T lymphocytes, *APCs* Antigen-presenting cells, *MDSCs* Myeloid-derived suppressor cells, *TILs* Tumor-infiltrating lymphocytes

DCs also express TIM-3, with Type 1 conventional DC (cDC1) exhibiting the highest level of TIM-3 expression [87, 88]. The binding PtdSer to TIM-3 efficiently facilitates the removal of apoptotic cells, thereby enabling TIM-3-positive DCs to engage in antigen cross-presentation [44]. The interaction betweenTIM-3 and the HMGB-1 mediates the suppression of tumor-infiltrating DCs by competing with nucleic acid for binding to HMGB-1 [35]. Additionally, TIM-3 promotes the polarization of M2-like macrophages [11, 80–82, 89]. Research has demonstrated that suppressing the TIM-3/Gal-9 signaling can effectively restrain macrophage M2 polarization in glioblastoma models [81].

The cytotoxic capability and IFNγ production exhibited by NK cells are essential to effectively combat cancers. TIM-3 is constitutively expressed on NK cells and is especially abundant in mature CD56^dim^CD16^+^ NK cells, making it a reliable marker of maturity [28]. The elevated expression of TIM-3 enhances the NK cell’s cytotoxic activity; however, prolonged stimulation can lead to abnormal or excessive TIM-3 expression, resulting in a subset of exhausted or dysfunctional NK cells [90]. Therefore, TIM-3 is used as a marker for exhaustion in cancer-infiltrating NK cells, and its blockade effectively restores their functionality [16]. Although the precious role of TIM-3/Gal-9 pathway in NK cells remains largely unexplored, blocking this pathway can revive their impaired function. Research has shown that engagement of TIM-3 with agonist antibodies or target cells expressing Gal-9 significantly reduces the cytotoxicity produced by the NKL (a human NK-cell line) and human primary peripheral blood NK cells [28, 78]. TIM-3 inhibition has been shown to increase peripheral blood NK cell’s cytotoxic activity to kill K562 target cells [91], as well as to boost effector activity in patients with bladder cancer.

### PD-1

PD-1 (CD279), which *PDCD1* encodes, was first isolated in 1992 [92]. PD-1 belongs to CD28 superfamily [93]. PD-1 expresses in various immune cell types, including T cells, DCs, B lymphocytes, NK cells, macrophages, monocytes, and myeloid cells. It delivers inhibitory signals by interacting with its ligands, which is important for avoiding over-activation and reducing the chances of autoimmune diseases [94]. PD-1 expression is upregulated following the activation of T cells. Tumors use the PD-1-mediated inhibitory pathway to reduce anti-tumor immunity and avoid immune system destruction, which increases tumor survival and growth [95, 96]. PD-1 signaling induces T cell exhaustion, and the inhibition of PD-1 can effectively reverse this exhaustion, thereby restoring a normal anti-tumor immune response [97]. This treatment method has proven to be effective in cancer immunotherapy.

#### Structure of PD-1 and its ligands

There are 288 amino acid residues in both human and mouse PD-1. There is a 60.1% homology between the two species. PD-1is a type I transmembrane protein, characterized by an extracellular immunoglobulin variable (IgV)-like domain, a hydrophobic region across the membrane, and an intracellular tail in the cytoplasm. PD-1 contains two common inhibitory motifs known as ITIM (VDY223GEL) and ITSM (TEY248ATI) in its cytoplasmic tail [42, 51], functioned as possible phosphorylation sites [98]. The activated ITSM is crucial in suppressing T cell function [99, 100].

Two ligands are recognized by PD-1’s IgV-like domain, namely PD-L1 and PD-L2 (Table 1), both of which are members of the B7 family. PD-L1 was initially found in 1999 [101] and serves as PD-1’s main ligand. The *PDCDL1* gene encodes this type I transmembrane protein. PD-L1 contains two extracellular structural domains, IgV and IgC [96]. Numerous tissues express PD-L1, including the lungs, heart, placenta, and skeletal muscles [101]. T cells, DC cells, B cells, macrophages, non-hematopoietic cells like keratinocytes, and non-parenchymal cells constitutively express PD-L1 [101, 102]. Researchers have discovered that PD-1 receptor on T cells can engage with the PD-L1 on cancer cells. Because of this interaction, T cell activation is suppressed [103] and apoptosis is induced [104]. This process consequently suppresses anti-tumor immunity [95, 105]. PD-L2 displays a more limited expression pattern and primarily expressed by APCs [106]. It exhibits a higher binding affinity for PD-1.

#### Signal transduction of PD-1

Upon interaction with PD-L1 or PD-L2, the tyrosine residue in PD-1’s ITSM became phosphorylated, leading to recruitment of Src homology 2 domain-containing protein tyrosine phosphatase 2 (SHP-2). SHP-2 is a key dephosphorylase. It subsequently dephosphorylates of essential molecules involved in TCR and CD28 signals pathways, such as CD3ε and ZAP70. Consequently, this inhibits both TCR and CD28 signaling, eventually suppressing T cell-associated signaling (Fig. 2b) [51, 107–109]. Through SHP2, PD-1 exerts a directly inhibitory effect on PI3K-AKT signaling, leading to reduction in T cell activation [110–113]. Additionally, SHP2 dephosphorylates the cytoplasmic tail of CD28, thereby counter-regulating PI3K-AKT activity [109].

Furthermore, SHP2 prevents RAS activation, which subsequently suppresses the RAS-MEK-ERK pathway, thereby inhibiting T cell proliferation [114, 115]. The primary mechanisms by which PD-1 inhibits the RAS-MEK-ERK cascade are dephosphorylation of PLCγ1 and direct inhibition of RAS. SHP-2 also inhibits protein kinase C-theta (PKCθ), which causes PKCθ-mediated NFκB activity to be downregulated [51, 116]. Moreover, PD-1 ligation inhibits the production of IL-2 and interferon, as well as the expression of the cell survival factor B-cell lymphoma-extra-large (Bcl-xL) in T cells, which affects cellular survival and proliferation [110, 117]. These modifications ultimately result in the suppression of immune effector cells’ cytotoxic activity, cell proliferation, and cytokine generation, thereby facilitating the tumor evasion from the immune system. PD-1/PD-L1 inhibitors block the interaction between PD-L1 and PD-1, thereby restoring the effector function of T cells [118].

#### The physiological function and roles of PD-1 in immune cells

The inhibitory signals conveyed by PD-1during its interaction with PD-L1 or PD-L2 are crucial for the regulation of T cell activation. This signaling is essential not only for facilitating immune tolerance but also for maintaining immune homeostasis. Under typical physiological conditions, PD-L1 and PD-1 play pivotal roles in maintaining this intricate equilibrium within the immune system [95]. Their engagement prevents excessive autoimmune responses against self-tissues and matains immune equilibrium by suppressing T cell hyperactivation [119, 120]. PD-1/PD-L1 axis disruption has been associated with autoimmune diseases in both murine models [121, 122] and humans [123, 124].

Activated T cells, NK cells, B cells, macrophages, DC cells, monocytes cells, and tumor-specific T cells express PD-1, therefore it is essential for suppressing both innate and adaptive immune responses. PD-1 inhibitors mainly modulate the activity of the CTLs. When PD-L1 binds CD8^+^ T cell's PD-1 receptors, downstream signaling is activated, which eventually results in T cell exhaustion or death [125]. DCs are essential to effectiveness of anti-PD-1 treatment [126, 127].

## The function of TIM-3 and PD-1/PD-L1 in cancer immune evasion

The TME plays a pivotal role in influencing tumor development and progression. Immune dysfunction, which results from intricate interactions between the immune system and malignant cells, helps create a TME that enables cancer cells to evade immune surveillance. Within this context, PD-1 and TIM-3 play crucial roles in controlling immunological exhaustion [128]. Beyond their extrinsic roles, the functional significance of PD-1/PD-L1 and TIM-3’s intrinsic expression in cancer cells has been investigated. This section will explore the function of TIM-3 and PD-1 in T cell exhaustion as well as their internal effects on cancer cells.

### TIM-3 and PD-1 mark different stages of exhausted T cell

**Fig. 3:**
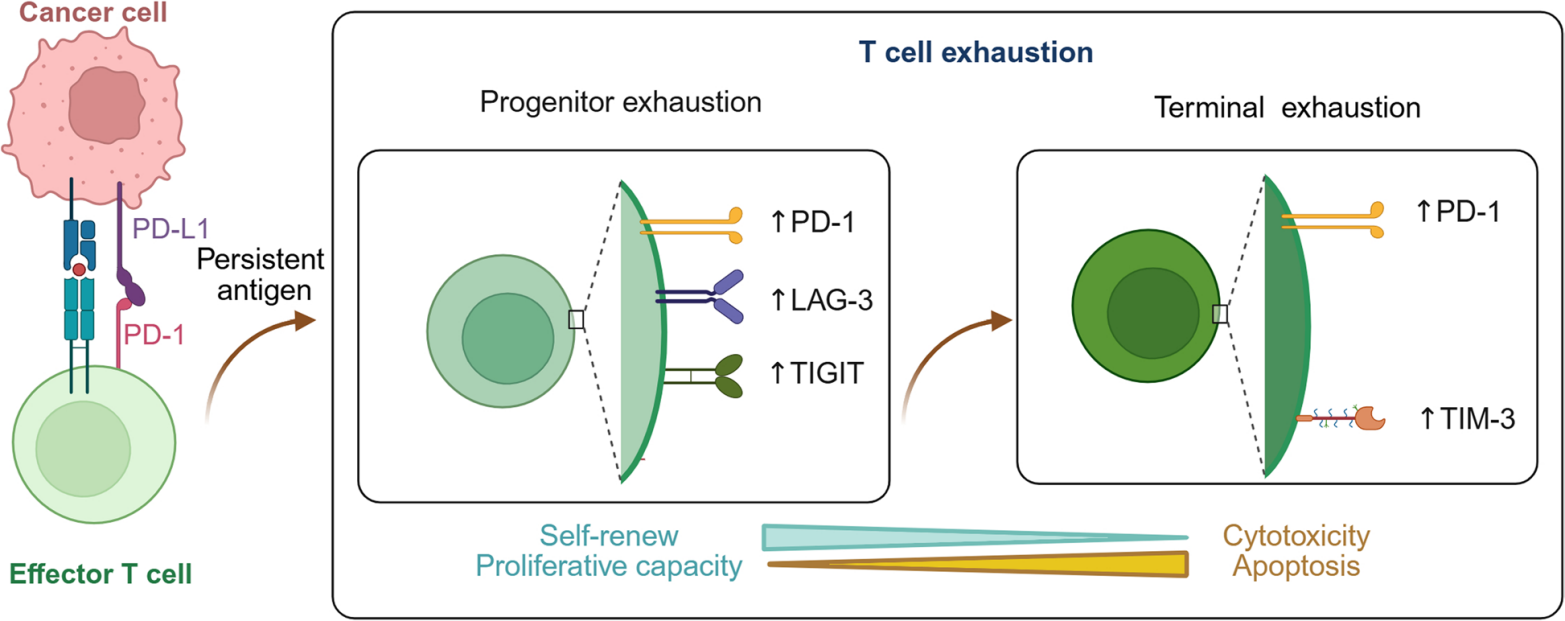
Exhausted T cells in cancer. T cell exhaustion is characterized by a progressive decline in proliferative capacity, the upregulation of co-inhibitory molecules (such as PD-1, TIM-3, LAG-3, and TIGIT), and an increased rate of apoptosis. Two distinct subsets of exhausted CD8^+^ T cell have been identified. Progenitor exhausted T cells are distinguished by their enhanced proliferative capacity, self-renew ability, production of polyfunctional cytokines, long-term persistence, and potential to differentiate into terminally exhausted cells. Terminally exhausted T cells exhibit enhanced cytotoxicity but possess diminished proliferative potential, polyfunctional cytokines, and longevity

In response to an acute immunological assault, CD8^+^ T cells typically exhibit a transitory response. After antigen clearance, most effector cells undergo apoptosis, while a small percentage differentiate into a population with long-lasting memory [129]. Prolonged expose to high antigen level results in sustained CD8^+^ T cell activation, ultimately resulting in their exhaustion [130]. This state of is characterized by restricted proliferation ability, elevated levels of inhibitory receptors (PD-1, TIM-3, TIGIT, and LAG-3), weakened effector function, and increased susceptibility to apoptosis (Fig. 3) [131].

Tumor progression is mainly caused by the exhaustion of TILs [129]. The overexpression of TIM-3 and PD-1 within the TME profoundly influences tumor-specific T cell response. Usually, the PD-1 typically inhibits CD8^+^ T cells from responding to neoantigens, thereby enables cancer cells to evade the cytotoxic effects of the immune system [132]. Studies have shown that specific subsets of T cells in AML, particularly CD8^+^ T cells, exhibit increased PD-1 and TIM-3 expression [133, 134]. The elevated presence of PD-1^+^TIM-3^+^CD8^+^ T cells may be associated with T cell dysfunction in AML, potentially influencing patient clinical outcomes [135]. Additionally, TILs found in various solid tumors, including melanoma, breast cancer, ovarian cancer, and colorectal cancer, exhibit significantly elevated levels of PD-1 and TIM-3 [136–139].

Recently, two different subgroups of exhausted CD8^+^ TILs have recently been found: progenitor exhausted CD8^+^ T cells and terminally exhausted CD8^+^ T cells [140, 141] (Fig. 3). Progenitor exhausted CD8^+^ T cells, commonly known as stem-like exhausted CD8^+^ T cells (characterized by PD-1^int^Slamf6^+^ or CXCR5^+^), are identified by their capacity to produce polyfunctional cytokines, enhances proliferative capacity, long-term persistence, and their capacity to differentiate into terminally exhausted cells [140, 142, 143]. This subpopulation is specifically targeted by PD-1-targeted immunotherapy, which promotes their differentiation into effector cells by inducing proliferation [141, 143].

Terminally exhausted CD8^+^ T cells (PD-1^hi^TIM-3^+^) demonstrate enhanced cytolytic activity, despite their diminished capacity for polyfunctional cytokine production, proliferation, and longevity [142, 143]. TIM-3 identifies the most dysfunctional subset of infiltrating PD-1^+^CD8^+^ T cells within tumors [144], highlighting its crucial role in mediating immunosuppression. Tim-3^+^PD-1^+^ tumor-reactive CD8^+^ T cells are prevalent in TILs [145]. Additionally, TIM-3 has been identified as a potential negative prognostic indicator in human malignant tumors [146]. Increased levels of TIM-3 on CD8^+^ T cells are associated with tumor progression and correlate with a poor prognosis [147–150].

When anti-TIM-3 mAbs were initially administered to mice with B16 melanoma and CT26 CC (CT26 and MC38 models) in 2010, their efficacy was found to be limited [144, 151]. However, the combination of TIM-3-Ig with an anti-PD-L1 mAb showed impressive anticancer effects [144]. In mouse models of chronic myeloid leukemia, the simultaneous blockade of TIM-3 and PD-1 effectively relieved CD8^+^ T cell exhaustion [152, 153].

### Cancer cell-intrinsic TIM-3 expression

Cancer has been linked to increased levels of TIM-3 and its ligand Gal-9 [154–156], which promotes the establishment of a TME that suppresses immune cell activity within the tumor. Besides immune cells, hematopoietic stem cells in myelodysplastic syndromes (MDS) [157] and leukemic stem cells (LSC) in acute myeloid leukemia (AML) can express TIM-3 [20, 158, 159]. In a recent study including 302 AML patients, TIM-3 was detected in LSCs in 78.5% of cases at the time of first diagnosis [22, 160]. TIM-3 up-regulation is linked to a poor prognosis in AML [160, 161]. However, the precious mechanism by which TIM-3 affects the prognosis of AML is not well understood [160]. It has been proposed that Gal-9, which is released by TIM-3^+^ AML cells, enhances the LSCs self-renewal through a feedback mechanism [159]. NFκB and β-catenin signaling pathways, both recognized for their roles in supporting LSC self-renewal, were co-activated by Gal-9 mediated activation of TIM-3 [162].

TIM-3 is also expressed on a variety of cancer cells in solid tumors [151], including melanoma [36, 163, 164], colon carcinoma [165], hepatocellular carcinoma cells (HCC) [166, 167], osteosarcoma [168], and non-small cell lung cancer (NSCLC) [169]. All of these cancers, excluding melanoma, have more aggressive disease development linked to elevated levels of TIM-3 expression. For instance, tumor cell-intrinsic TIM-3 stimulates the growth of liver cancer cells via the NFκB/IL-6/STAT3 signaling pathway [167]. Anti-TIM-3 antibodies can inhibit tumor growth by blocking TIM-3 on malignant hepatocytes in vitro and in TIM-3 knockout mouse models [167].

In contrast, TIM-3 acts as an intrinsic growth-suppressive receptor in melanoma cells [164]. Enhanced tumor growth was linked to TIM-3 inhibition in melanoma cells in both immunocompetent and immunodeficient mouse models. Conversely, overexpression of TIM-3 specific to melanoma cells reduced tumorigenesis. Furthermore, administration of the TIM-3 antibody enhanced tumor formation in both murine and human melanomas with high and low immunogenicity in T-cell-deficient mice, which verified that antagonizing TIM-3 on melanoma cells can promote tumor growth.

### Cancer cell-intrinsic PD-1/PD-L1 expression

#### Cancer cell-intrinsic PD-L1

Most cells usually have low levels of PD-L1 expression in normal physiological states. However, its expression is remarkably enhanced on the cancer cell surface. A wide diversity of tumor cells expresses PD-L1, including those from leukemia, renal cell carcinoma, melanoma, glioma, breast cancer, and NSCLC [104, 170, 171]. The majority of researchers have focused on the typical cell-extrinsic function of PD-L1 as PD-1’s ligand. When PD-L1 on tumor cells engages with PD-1 on TILs, an inhibitory signal is conveyed to T cells. This interaction disrupts both the TCR signaling cascade and the B7/CD28 co-stimulatory pathway. This ultimately leads to impaired effector T cell function and even exhaustion. Consequently, this mechanism enables tumor cells to avoid immune surveillance [95, 172].

By engaging in certain oncogenic pathways, PD-L1 has recently been demonstrated in an increasing number of studies to have tumor-intrinsic functions in cancer development and chemoresistance independent of PD-1. Its intrinsic functions include cancer cell survival, stemness, invasion, chemotherapy resistance, glycolysis, interferon response pathways and DNA damage response [173–180]. Multiple signaling pathways regulate PD-L1expression in cancer cells, including NFκB, mTOR, STAT, MAPK, and c-Myc [181].

PD-L1 can stimulate intrinsic signaling to increase bladder cancer cell survival and proliferation through mTOR pathway activation and autophagy inhibition [182]. PD-L1 plays a significant role in the tumorigenesis and spread of ovarian cancer [183–185]. By engaging in the c-JUN/VEGFR2 signaling pathway, PD-L1 promoted the dissemination and angiogenesis of ovarian cancer [185]. The anti-PD-L1 mAb durvalumab and anti-angiogenic drug apatinib combination has the potential to strengthen the anti-angiogenesis effect as well as the suppression of invasion and cell migration [185].

It has been observed that cancer intrinsic PD-L1 accelerates the epithelial-mesenchymal transition (EMT) in ovarian cancer, melanoma, breast cancer, nasopharyngeal carcinoma, and lung cancer [173, 177, 179, 186, 187]. EMT is recognized as a critical process closely linked to the resistance to treatment and progression of cancer. PD-L1 promotes EMT by preventing riple-negative breast cancer (TNBC) cells from deleting the EMT transcription factor Snail. PD-L1 antibodies can restrain the development of TNBC in immunodeficient animals by blocking the PD-L1/Snail pathway [177]. Moreover, PD-L1’s intrinsic function was linked to resistance to anti-PD-1 therapy in models of murine colon cancer and melanoma [188, 189].

#### Cancer cell-intrinsic PD-1

In addition to immune cells, tumor cells also express PD-1 [190]. The cancer cell-intrinsic PD-1 was initially identified in melanoma [186, 191–196]. Later, the intrinsic expression of PD-1 in tumor cells has also been identified in HCC [197, 198], NSCLC [199, 200], colorectal carcinomas [201, 202], Merkel cell carcinoma (MCC) [203], pancreatic cancer [204], and glioblastoma [205]. The role of cancer intrinsic PD-1 varies across different tumor types [206, 207]. It remains unknown how the intrinsic PD-1 gene and protein expression are regulated in cancers and how this affects ICIs [196].

Cancer cell-PD-1 exhibits growth-inhibitory activities in NSCLC [199, 200] and colorectal carcinoma [201, 202]. In vitro, mouse lung cancer cell line M109 viability was improved by PD-1 knockout or antibody blockage, and in immune-deficient animals, M109-xenograft tumors developed more quickly as a result of PD-1 suppression, exhibiting enhanced proliferation and reduced apoptosis [199]. When PD-1 was expressed by human colon cancer cells, internal PD-1 signaling dramatically inhibited proliferation and boosted apoptosis [201]. On the contrary, anti-PD-1 antibody nivolumab increased proliferation, cut down apoptosis, and protected PD-1^+^ cells from chemotherapy and radiotherapy [201].

In addition, cancer cell-intrinsic PD-1 has been shown to promote tumorigenesis in various cancers, including melanoma [186, 191, 195], MCC [203], HCC [197], pancreatic cancer [204], and glioblastoma [205]. Recent research has shown that intrinsic-PD-1 can boost the development of MCC by triggering the downstream mTOR-mitochondrial ROS signaling pathway. On the contrary, antibody-mediated blockade of PD-1 has been demonstrated to inhibit this process [203].

## Therapeutic strategies targeting PD-1 and TIM-3

### Current PD-1/PD-L1 inhibitors

PD-1/PD-L1 inhibitors disrupt the interaction between PD-1 and PD-L1 by specifically binding to either of these molecules. By obstructing the downstream inhibitory signals mediated by PD-1, this interference enables immune cells to identify and eliminate tumor cells, thereby preventing them from evading immune surveillance. PD-1/PD-L1 inhibitors offer long-lasting therapeutic effects and improve overall patient survival in a variety of cancer types. Anti-PD-1 antibodies (pembrolizumab, nivolumab, cemiplimab, dostarlimab, and tislelizumab) and anti-PD-L1antibodies (atezolizumab, durvalumab, and avelumab) have received authorization from the FDA for the treatment of various hematologic and solid cancers [208, 209] (Table 3). Additionally, the global approval of other PD-1 blocking antibodies such as camrelizumab, toripalimab, sintilimab, zimberelimab, and prolgolimab has further expanded their application in cancer therapy [208, 209].

**Table 3:** The details of the FDA-approved inhibitors of PD-1/PD-L1

Target	Drugs(Brand name)	Description	Manufacturer	Time of approval	Cancer type
PD-1	Pembrolizumab (Keytruda)	Humanized IgG4	Merck	2014	Melanoma; NSCLC; HNSCC; cHL; Gastric cancer; Cervical cancer; HCC; MCC; CRC; ES-SCLC; RCC; EC; TMB-high solid cancers; TNBC; CSCC; UC; Primary mediastinal large B-cell lymphoma; ESCC
	Nivolumab (Opdivo)	Human IgG4	Bristol-Myers Squibb	2014	Melanoma; NSCLC; RCC; cHL; HNSCC; UC; Gastric cancer; HCC; CRC; Malignant pleural mesothelioma; ESCC; OC
	Cemiplimab (Libtayo)	Human IgG4	Regeneron Pharmaceuticals	2018	CSCC; NSCLC; Basal cell carcinoma; Cervical cancer
	Dostarlimab (Jemperli)	Humanized IgG4	GlaxoSmithKline LLC	2021	EC; dMMR solid cancers
	Tislelizumab (Tevimbra)	Humanized IgG4	BeiGene	2023	ESCC; NSCLC
PD-L1	Atezolizumab (Tecentriq)	Human IgG1	Genentech/Roche	2016	UC; NSCLC; TNBC; HCC; ES-SCLC
	Durvalumab (Imfnzi)	Humanized IgG1	AstraZeneca	2017	UC; NSCLC; ES-SCLC; HCC
	Avelumab (Bavencio)	Humanized IgG1	Merck	2017	MCC; UC; RCC; OC

*FDA* Food and Drug Administration, *PD-1* Programmed cell death protein 1, *PD-L1* Programmed death-ligand 1, *HNSCC* Head and neck squamous cell carcinoma, *NSCLC* Non-small cell lung cancer, *cHL* classic Hodgkins Lymphoma, *HCC* Hepatocellular carcinoma, *MCC* Merkel cell carcinoma, *CRC* Colorectal cancer, *ES-SCLC* Extensive stage small cell lung cancer, *RCC* Renal cell, *EC* Endometrial Carcinoma, *TMB* Tumor mutational burden, *TNBC* Triple negative breast cancer, *CSCC* Cutaneous squamous-cell carcinoma, *UC* Urothelial carcinoma, *ESCC* Esophageal squamous cell carcinoma, *OC* Ovarian cancer, *dMMR* deficient mismatch repair

Although PD-1/PD-L1inhibitors demonstrate potent anti-tumor efficacy in certain patients, several challenges remain. These challenges include primary and acquired drug resistance, which limits the therapeutic benefits for the majority of patients [210]. The major immunological resistance mechanisms to anti-PD-1/PD-L1 treatment encompass the presence of immunosuppressive components in the TME, the lack of a PD-L1 target, and T cell exhaustion resulting from the upregulation of alternative immune checkpoints [211–215].

### Development of TIM-3 inhibitors

By directly suppressing TIM-3 function in tumor cells and modulating the activity of various immune cell types, TIM-3 blockage can promote tumor regression and the formation of anticancer immunological memory [19]. Currently, a wide array of TIM-3-targeted immunotherapeutic agents are undergoing clinical trials in phase 1 / 2. These agents include Cobolimab, Sabatolimab, BGB-A425, BC3402, TQB2618, NB002, AZD7789, LB1410, and INCAGN02390. The characteristics of these agents are concisely presented in Table 4. The first-in-class TIM-3 antibody, sabatolimab (MBG453), a humanized IgG4 antibody, exhibits high affinity for specific binding to TIM-3 and effectively inhibits its interaction with Gal-9 and PtdSer [4, 110].

**Table 4:** Anti-human TIM-3 antibody agents under clinical investigation

Drug	Description	Manufacturer
Sabatolimab (MBG453)	mAb, humanized IgG4 (S228P; stabilized hinge mutation) mAb	Novartis Pharmaceutical
Cobolimab (TSR-022)	mAb, humanized IgG4 (S228P)	Tesaro
BGB-A425	mAb, humanized IgG1	BeiGene
BC3402	mAb, humanized IgG4	BioCity
TQB2618	mAb, humanized IgG4	Chiatai Tianqing (CTTQ)
NB002	mAb, humanized IgG1	NeoLogics Bioscience
AZD7789	Bispecific antibody to PD-1/TIM-3	AstraZeneca
LB1410	Bispecific antibody to PD-1/TIM-3	L&L Biopharma
INCAGN02390	mAb, humanized IgG1κ, N297A (Fc-engineered silent)	Incyte, Agenus

Sabatolimab has demonstrated immuno-myeloid activity within a potential dual mechanism against AML and myelodysplastic syndrome (MDS) through reactivating the immune system to eliminate LSCs and blasts, as well as directly targeting TIM-3 on leukemic blasts to inhibit cancer cell proliferation [216]. Sabatolimab in combination with hypomethylating agent (HMA) was administrated to 53 patients having high- or very high-risk MDS (HR/vHR-MDS) and 15 patients with chronic myelomonocytic leukemia (CMML) in the STIMULUS clinical trial (NCT03066648) [217]. The most frequent adverse effects (AE) observed in HR/vHR-MDS patients included thrombocytopenia (56.6%), constipation (56.6%), and nausea (54.7%). Immune-related adverse events (AEs) were observed in seven patients. The median duration of response (mDOR) was 17.1 months, and the overall response rate (ORR) was 56.9% among the 51 HR/vHR-MDS evaluation patients. The mDOR for CMML patients was 5.6 months, and the ORR was 66.7%.

## Synergistic effects and mechanisms of TIM-3 and PD-1 inhibitors

### TIM-3 and PD-1 inhibitors synergistically activate CD8^+^ T cells

PD-1 inhibitor therapy results in the upregulation of TIM-3 in a variety of cancer types [3, 9–12], which is recognized as a mechanism contributing to resistance against anti-PD-1 treatment. Among 16 patients with metastatic melanoma who were treated with pembrolizumab monotherapy, non-responders demonstrated significantly elevated TIM-3 expression on their CD8^+^ T cells [218, 219]. Additionally, PD-1 has the ability to bind to Gal-9, which reduces cell death caused by the Gal-9/TIM-3 interaction and promotes the survival of PD-1^+^TIM-3^+^ T cells [24]. TIM-3 and PD-1 are widely acknowledged as critical biomarkers of T cell exhaustion [220]. PD-1^+^TIM-3^+^CD8^+^ TILs showed more exhaustion and stronger suppression capabilities compared to PD-1^+^Tim-3^−^CD8^+^ T cells [148, 221–223]. Studies have confirmed in glioblastoma, a low number of CD8^+^CD103^+^PD1^+^TIM3^+^ memory T cells are the most predictive independent indicator related to longer OS [224]. Additionally, CD4^+^ and CD8^+^ T lymphocytes isolated from HCC tissues demonstrated elevated PD-1 and TIM-3, which are linked to unfavorable outcomes [225].

TIM-3 inhibitors enhance the functionality of effector T cells by disrupting the interaction between TIM-3 and its ligands. When Gal-9 on tumor cells interacts with TIM-3 on T cells, it effectively suppresses T cell activation and even induces their apoptosis. This interaction consequently reduces cytokine production and facilitates immune evasion by tumor. Anti-TIM-3 mAb can inhibit TIM-3/Gal-9 signaling, thereby restoring the activity of effector T cells. This restoration ultimately leads to an enhanced production of interleukin 2 (IL2) and IFNγ [147]. Anti-TIM-3 antibodies have been shown to suppress tumor development and enhance IFNγ-mediated antitumor immunity in preclinical cancer models [15]. Anti-TIM-3 antibodies have also been reported to work by preventing TIM-3 from interacting with PtdSer and CEACAM-1 [48]. Additionally, research has shown that anti-TIM-3 antibody M6903 effectively prevents Gal-9, PtdSer, and CEACAM-1from binding to TIM-3 [226]. According to our earlier research, anti-TIM-3 antibodies that recognize conformational epitopes on TIM-3 rather than linear epitopes hindered the interaction between TIM-3 and Gal-9 [227].

Recently, small molecule inhibitors targeting TIM-3 have demonstrated promising anti-tumor efficacy in preclinical studies. Specifically, ML-T7, which interacts with the FG-CC’ loop of TIM-3 to disrupt the binding of PtdSer and CEACAM1, has been demonstrated to improve both the survival and antitumor efficacy of primary CD8^+^ CTLs [228]. Furthermore, it has been found that ML-T7 enhances the cytotoxicity of NK cells and improves the antigen presentation capability of DCs. Additionally, SMI402, which effectively inhibits TIM-3 from binding to CEACAM-1, HMGB-1, and PtdSer [229], has demonstrated its capacity to suppress tumor growth by promoting the infiltration and activation of CD8^+^ T cells and NK cell at the tumor site [229].

A synergistic effect has been shown in the concurrent targeting of the TIM-3 and PD-1 pathways, as evidenced by the reactivation of T cells in vitro and the induction of anti-cancer responses in animal models [144, 221]. PD-1 and TIM-3 blockade significantly enhanced the expansion of tumor antigen-specific CD8⁺ T cells induced by melanoma vaccines, as well as their cytokine production [230]. When anti-PD-1 and anti-TIM-3 mAbs were administrated in combination, the median survival in a murine glioblastoma model increased significantly from 33 to 100 days. Moreover, this combined treatment significantly elevated the OS rate from 27.8% to 57.9% [231]. In a xenograft mouse model of HCC, the dual inhibition of TIM-3 and PD-1 demonstrated a significantly enhanced anti-tumor effectiveness compared to monotherapy [225]. This combination therapy not only reduced the expression level of PD-1 and TIM-3 on CD8^+^ T cells within tumor tissues but also enhanced T cell infiltration into tumors. Moreover, it led to a rise in the production of effector cytokines TNFα and IFNγ, while simultaneously reducing the levels of immunosuppressive cytokines IL6 and IL10 in tumor tissues.

The synergistic interactions between TIM-3 and PD-1/PD- L1 inhibitors in T cells are depicted in Fig. 2. Antibodies that target at PD-1/PD-L1 inhibit the interaction between PD-L1 and PD-1, thereby hindering Lck from phosphorylating the intramembrane domain of PD-1, which impairs cell recruitment to SHP-2. Consequently, PD-1 inhibitors upregulate TCR, CD28, PI3K-AKT, RAS-MEK-ERK, and PKCθ-mediated NFκB activity pathways. It has been demonstrated that anti-PD-1 therapy raises TIM-3 via the PI3K-AKT pathway [9], leading to adaptive resistance to this treatment. Anti-TIM-3 antibodies can prevent PD-1 resistance [11]. In the presence of TIM-3 inhibitor, Itk-mediated phosphorylation of the intramembrane motif of TIM-3 is inhibited, preventing the release of Bat3 and cell recruitment to Fyn. Consequently, Bat3 recruits Lck, which then phosphorylates TCR and CD28, restoring their activation. The integration of TIM-3 and PD-1/PD-L1 inhibitors improves the transmission of activation signals to downstream proteins and signaling pathways, which strengthens T cells’ immunological response.

### TIM-3 and PD-1 inhibitors cooperatively enhance multiple processes in cancer-immune cycle

The cancer-immune cycle provides a conceptual framework that enhances our understanding of the sequence of events triggering anti-cancer immune responses [232, 233]. This process comprises seven distinct steps: (1) release of cancer cell-derived antigens (cancer cell death), (2) cancer antigen presentation by DCs/APC, (3) priming and activation of APC and T cells, (4) trafficking of CTLs to tumors, (5) infiltration of T cells into stroma and tumors, (6) recognition of cancer cells by T cells, and (7) killing of cancer cells (immune and cancer cells).

**Fig. 4:**
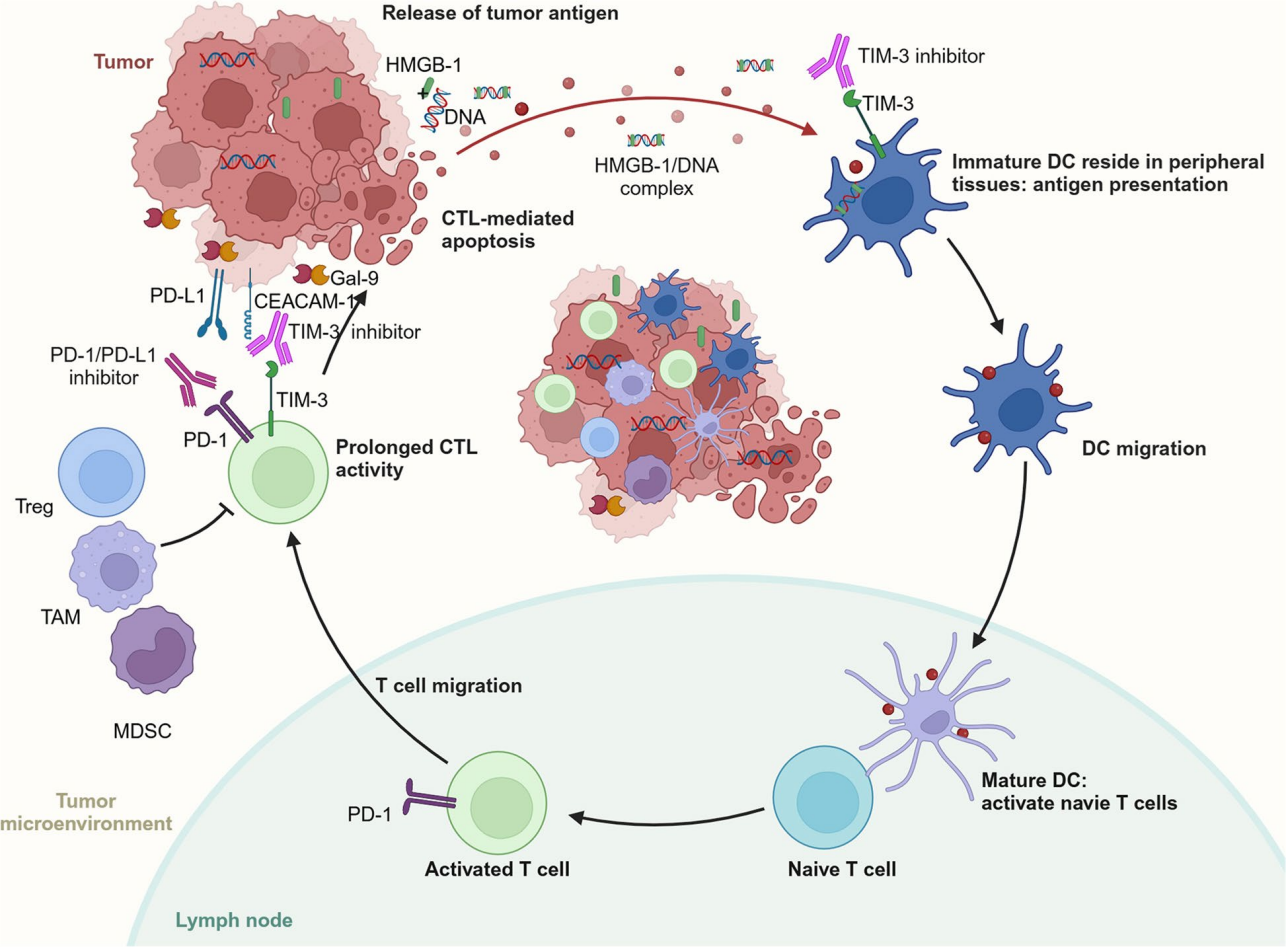
Essential function of TIM-3 and PD-1/PD-L1 inhibitors in cancer-immune cycle. TIM-3 inhibitors interfere with the binding of Gal-9, HMGB-1, or CEACAM-1 to TIM-3, thereby regulating the activity of cytotoxic T lymphocyte (CTL) and dendritic cell (DC). Meanwhile, PD-1/PD-L1 inhibitors primarily enhance the function of CTL by obstructing the interaction between PD-1 and PD-L1. MDSC: myeloid-derived suppressor cells; TAM: tumor-associated macrophage; Treg: regulatory T cell; HMGB-1: high-mobility group box 1 protein; CEACAM-1: carcinoembryonic antigen-related cell adhesion molecule 1. The schematic illustration is sourced from app.Biorender.com

Due to the differential regulation of receptor and ligand expression in both temporal and spatial dimensions during the immune response, TIM-3 and PD-1 exhibit distinct contributions. The simultaneous administration of TIM-3 and PD-1 inhibitors enhances various processes within the cancer-immunity cycle and modifies TME. Myeloid-derived suppressor cells (MDSCs), and tumor-associated macrophage (TAM), and Treg all aid in immune suppression in the TME. Figure 4 illustrates how these inhibitors regulate T cell priming and effector function in the TME at critical stages of the cancer-immune cycle. The anti-tumor response is improved when TIM-3 and PD-1/PD-L1 inhibitors collaborate to regulate CTLs and specific immune populations. TIM-3 inhibitors primarily modulate the function of CTLs and DCs, whereas PD-1 inhibitors mainly influence CTL activity [234]. Recent results indicate that mouse γδ T cell subsets that generate IL-17A are differentially regulated by PD-1 and TIM-3 [33].

Accumulating evidence suggests that TIM-3 plays a pivotal role in regulation of antitumor immunity in DCs. Combining TIM-3 inhibition with chemotherapy significantly amplifies antitumor immune responses by augmenting CD103^+^DC’s production of the critical chemokine CXCL9 in a breast cancer model [235]. The function of CD8^+^ T cells can be indirectly activated by TIM-3 inhibition via the regulation of the activity of cDCs in the TME [84, 236]. Inhibiting TIM-3 can reverse tolerogenic function of DCs and enhance CXCL9 production, which recruits CD8^+^ effector T cells to combat tumors [237]. TIM-3 and PD-1 blockade has been shown to improve the survival of tumor-bearing mouse by boosting the antigen presentation of classical type 2 dendritic cells (imcDC2) and improving the tumor-killing function of CD8^+^ tissue-resident memory cells CD8^+^ T cells (CD8^+^TRMs) [238].

### Clinical implications of combination therapy with TIM-3 and PD-1 Inhibitors

Although the long-term benefits of PD-1/PD-L1 inhibitors are well-documented, only a minority (approximately 20%) of patients demonstrate a response to this single-agent therapy. The limitation has motivated research into combination therapies to enhance efficacy. The FDA has granted the use of anti-PD-1/PD-L1 in conjunction with chemotherapy, angiogenesis inhibitors, targeted treatment, and immunotherapy (CTLA-4 or LAG-3 inhibitors). Clinical research has demonstrated that in several malignancies, the combination of checkpoint inhibition with anti-CTLA-4 and anti-PD-1 can significantly increase in overall survival (OS). Additionally, this combination has higher response rates in comparison to monotherapy with either treatment alone [49, 239, 240]. PD-1 and CTLA-4 control T cell activation via different and non-overlapping mechanisms. These molecules exert their effects at various sites and time points throughout the T cell development process [234, 241, 242]. However, this combination therapy is linked to increased rates of immune-related toxicities that exceed those observed with individual agents [243, 244]. The synergistic effect of combining PD-1 and LAG-3 inhibitors has been demonstrated [245, 246]. Nevertheless, in comparison to PD-1 inhibitor monotherapy, this combination carries a higher risk of treatment-related toxicities [243].

TIM-3 is predominately expressed on terminally exhausted T cells in TME and often coexists with PD-1. Consequently, a potential limitation of TIM-3 and PD-1 monotherapy is its inability to completely activate these exhausted T cells. TIM-3 monotherapy exhibited moderate antitumor efficacy when compared to PD-1 monotherapy in a preclinical model. Co-targeting of the PD-1 and TIM-3 pathways has been shown to exhibit synergistic effectiveness [144, 221, 225, 231, 238], a wide safety profile, and high tolerability according to preclinical studies [7]. In clinic setting, most anti-TIM-3 antibodies are now being evaluated in conjunction with anti-PD-1/PD-L1 antibodies, anti-LAG-3 antibodies, and chemotherapy. In a phase I/II clinical research (NCT02608268), sabatolimab was evaluated for its safety and efficacy in patients with advanced solid tumors, both as monotherapy and in combination with spartalizumab (anti-PD-1 mAb, IgG4, S228P; PDR001). Although monotherapy with sabatolimab did not demonstrate significant antitumor activity, the combination therapy of sabatolimab and spartalizumab provided preliminary evidence of antitumor efficacy and exhibited favorable tolerability [2]. The most common AEs associated with the combination treatment included fatigue (15%), decreased appetite (8%), diarrhea (7%), rash (7%), elevated aspartate aminotransferase (6%), and nausea (6%) [2].

The human IgG1 mAb LY3321367 efficiently prevents TIM-3’s binding to PtdSer and partially blocks its interaction with Gal-9 [247]. The combination of LY3321367 and LY3300054 (anti-PD-L1 mAb) has demonstrated both safety and favorable clinical efficacy in treatment of MSI-H/dMMR tumors that have not been previously exposed to PD-1/PD-L1 inhibitors, as evidenced by a phase I clinical trial (NCT02791334) [247]. A phase I clinical study (NCT03099109) of LY3321367 revealed favorable pharmacodynamics and pharmacokinetics, as well as manageable safety profiles, in the treatment of refractory advanced solid tumors; however, its anticancer efficacy was only modest [248]. Due to unanticipated immunogenicity, the phase I trial (J1C-MC-JZDA, NCT03752177) of the bispecific antibody LY3415244, which targets both PD-L1 and TIM-3, was prematurely terminated [249]. Further investigation is essential for a thorough understanding of the implications associated with TIM-3 and PD-1 inhibition.

A comprehensive overview of registered clinical trials that combine anti-TIM-3 and anti-PD-1/PD-L1 antibodies is given in Table 5. This overview does not include clinical trials that have been terminated or withdrawn for any reason. All these trails are in phase 1/2. Cancer types include hematological malignancies and solid tumors. In these combinations, anti-TIM-3 antibodies include TSR-022, BGB-A425, BC3402, TQB2618, LB1410, or INCAGN02390; anti-PD-1 antibodies contain TSR-042, Nivolumab, Tislelizumab, Penpulimab, Toripalimab, Pembrolizumab, INCMGA00012, or Retifanlimab; anti-TIM-3/PD-1 BsAgs involve AZD7789 and LB1410; anti-PD-L1 antibodies include Durvalumab and TQB2450. Other related ICIs include anti-LAG-3 antibodies (TSR-033, INCAGN02385, or LBL-007) and anti-CTLA4 antibody (Tremelimumab). Additionally, TIM-3 and PD-1 inhibitor in combination with anti-CTLA4 (BC3402** + **Durvalumab + Tremelimumab) and anti-LAG-3 (TSR-022 + TSR-042 + TSR-033, Tislelizumab + BGB-A425 + LBL-007, INCAGN02385 + INCAGN02390 + INCMGA00012, or Retifanlimab + INCAGN02385 + INCAGN02390) are being explored in various trails.

**Table 5:** Ongoing clinical trials involving combination therapies targeting TIM-3 and PD-1/PD-L1

Drug	NCT code	Status	Phase	Tumor types	Target	Investigated Agents
TSR-022	NCT04139902	Recruiting	Phase 2	Melanoma stage III and IV	TIM-3PD-1	TSR-042 (Anti-PD-1; Dostarlimab) and TSR-042 + TSR-022 (anti-TIM-3, Cobolimab)
	NCT06238635	Recruiting	Phase 2	Cervical cancer Advanced cervical carcinoma Metastatic cervical cancer Recurrent cervical carcinoma	TIM-3PD-1	TSR-042 + TSR-022
	NCT02817633	Recruiting	Phase 1	Neoplasms	TIM-3 PD-1 LAG-3	TSR-022, TSR-022 + Nivolumab (Anti-PD-1), TSR-022 + TSR-042, TSR-022 + TSR-042 + TSR-033 (Anti-LAG-3), TSR-022 + TSR-042 + Docetaxel, TSR-022 + TSR-042 + Pemetrexed + Cisplatin, TSR-022 + TSR-042 + Pemetrexed + Carboplatin, and TSR-022 + Docetaxel
	NCT03680508	Active, not recruiting	Phase 2	Adult primary liver cancer Advanced adult primary liver cancer Localized unresectable adult primary liver cancer	TIM-3 PD-1	TSR-022 + TSR-042
	NCT03307785	Active, not recruiting	Phase 2	Neoplasms Metastatic cancer Advanced cancer Solid tumor NSCLC metastatic NSCLC stage IIIB NSCLC	PARA1/2 PD-1 VEGF TIM-3	Niraparib (PARP1/2 inhibitor) + TSR-042, TSR-042 + Carboplatin-Paclitaxel, Niraparib + TSR-042 + Bevacizumab (anti-VEGF), TSR-042 + Carboplatin-Paclitaxel + Bevacizumab, TSR042 + TSR-022 + Carboplatin-Paclitaxel, TSR-042 + Carboplatin-Paclitaxel, and TSR042 + TSR-022 + Carboplatin-Nab-Paclitaxel
BGB-A425	NCT05909904	Active, not recruiting	Phase 2	HNSCC HNC	PD-1 TIM-3 LAG-3	Tislelizumab (anti-PD-1), Tislelizumab + BGB-A425(anti-TIM-3), Tislelizumab + LBL-007 (anti-LAG-3), and Tislelizumab + BGB-A425 + LBL-007
BC3402	NCT06111326	Not yet recruiting	Phase 1/2	HCC	TIM-3 PD-L1	BC3402 (anti-TIM-3) + Durvalumab (anti-PD-L1)
	NCT06608940	Not yet recruiting	Phase 1/2	HCC	TIM-3 PD-L1 PD-1	BC3402** + **Durvalumab + Tremelimumab
TQB2618	NCT05834543	Recruiting	Phase 1/2	Advanced Esophageal Squamous Cell Carcinoma	TIM-3 PD-1	TQB2618 (anti-TIM-3) + Penpulimab (anti-PD-1) + Chemotherapy, Penpulimab + Chemotherapy, and TQB2618 + Penpulimab + TQB3617 capsules (BET inhibitor)
	NCT05783921	Recruiting	Phase 1/2	Recurrent Squamous Cell Carcinoma of the Head and Neck Metastatic Squamous Cell Carcinoma	TIM-3 PD-1	TQB2618 + Penpulimab + Chemotherapy (Paclitaxel + Cisplatin or Carboplatin), and Penpulimab + Chemotherapy (Paclitaxel + Cisplatin or Carboplatin)
	NCT06010901	Recruiting	Phase 1	Colorectal Cancer	TIM-3 PD-1	TQB2618 + Penpulimab + Anlotinib hydrochloride capsules, TQB2618 + Penpulimab, and TQB2618
	NCT05975645	Recruiting	Phase 1	Advanced HCC	TIM-3 PD-1	TQB2618 + Penpulimab + Anlotinib Hydrochloride Capsules
	NCT05645315	Unknown status	Phase 1/2	Advanced Solid Tumor	TIM-3 PD-L1	TQB2618 (Anti-TIM-3) + TQB2450 (Anti-PD-L1)
	NCT05563480	Unknown status	Phase 2	Nasopharyngeal Carcinoma	TIM-3 PD-1	TQB2618 + Penpulimab + Gemcitabine hydrochloride injection + cisplatin injection, Penpulimab + Gemcitabine hydrochloride injection + cisplatin injection, and TQB2618 + Penpulimab
	NCT05400876	Unknown status	Phase 1/2	Relapsed/Refractory Lymphoma	TIM-3 PD-1	TQB2618 + Penpulimab
	NCT05451407	Unknown status	Phase 1	Melanoma	TIM-3 PD-1	TQB2618 + Toripalimab (Anti-PD-1)
AZD7789	NCT05702229	Recruiting	Phase 2	Gastric Cancer	PD-1/CTLA4 PD-1/TIGIT TIM-3/PD-1 Claudin18.2	Volrustomig (PD-1/CTLA4 BsAb) + FOLFOX or XELOX, Rilvegostomig (PD-1/TIGIT BsAb) + FOLFOX or XELOX, Volrustomig + AZD0901 (anti-Claudin18.2 ADC) + 5-Fluorouracil or Capecitabine, Rilvegostomig + AZD0901 + 5-Fluorouracil or Capecitabine, AZD7789 + FOLFOX or XELOX, and AZD7789 + AZD0901 + 5-Fluorouracil or Capecitabine
	NCT04612751	Recruiting	Phase 1/2	Carcinoma, NSCLC Gastric cancer GEJC	TIM-3/PD-1	AZD7789 (TIM-3/PD-1BsAb)
	NCT06366451	Recruiting	Phase 1	HNSCC	PD-1/TIGIT PD-1/CTLA4 TIM-3/PD-1 HER2 PD-1	Rilvegostomig, Volrustomig, AZD7788, AZD9592 (anti-HER2 ADC), Pembrolizumab (anti-PD-1), AZD9592 + Rilvegostomig, AZD9592 + Volrustomig, AZD9592 + Sabestomig, and AZD9592 + Pembrolizumab
	NCT04541108	Recruiting	Phase 1	Solid tumor	PD-1/TIGIT PD-1/CTLA4 TIM-3/PD-1 PD-1	Rilvegostomig, Volrustomig, AZD7788, and Pembrolizumab
	NCT04931654	Active, not recruiting	Phase 1/2	Carcinoma, NSCLC Gastric Cancer GEJC	TIM-3/PD-1	AZD7789
	NCT05216835	Active, not recruiting	Phase 2	Relapsed or Refractory Classical Hodgkin Lymphoma	TIM-3/PD-1	AZD7789
LB1410	NCT06468358	Recruiting	Phase 1/2	Solid Tumor	TIM-3/PD-1 Claudin18.2/IL-10	LB1410 (TIM-3/PD-1 BsAb) + LB4330 (anti-claudin18.2/IL-10 fusion protein)
	NCT05357651	Recruiting	Phase 1	Solid tumor Lymphoma	TIM-3/PD-1	LB1410
INCAGN02390	NCT04370704	Active, not recruiting	Phase 1/2	Melanoma	TIM-3 LAG-3 PD-1	INCAGN02385 (Anti-LAG-3) + INCAGN02390 (Anti-TIM-3) and INCAGN02385 + INCAGN02390 + INCMGA00012 (Anti-PD-1)
	NCT05287113	Active, not recruiting	Phase 2	HNC	PD-1 LAG-3 TIM-3	Retifanlimab (Anti-PD-1), Retifanlimab + INCAGN02385, and Retifanlimab + INCAGN02385 + INCAGN02390
	NCT06056895	Active, not recruiting	Phase 2	Unresectable Clinical Stage III MCC AJCC v8 Clinical Stage IV MCC AJCC v8 MCC	PD-1 LAG-3 TIM-3	Retifanlimab + INCAGN02385 + INCAGN02390
	NCT04463771	Active, not recruiting	Phase 2	Endometrial Cancer	PD-1 IDO1 LAG-3 TIM-3	Retifanlimab, Retifanlimab + Epacadostat (IDO1 inhibitor), Retifanlimab + Pemigatinib (Anti-FGFR), and Retifanlimab + INCAGN02385 + INCAGN02390

Data source: https://clinicaltrials.gov/. *PD-1* Programmed cell death protein 1, *PD-L1* Programmed death-ligand 1, *LAG-3* Lymphocyte activation gene 3, *PARP1/2* Poly ADP-ribose polymerase 1/2, *VEGF* Vascular endothelial growth factor, *CTLA4* Cytotoxic T-lymphocyte associated antigen 4, *TIGIT* T cell immunoreceptor with immunoglobulin and ITIM domain, *BsAb* Bispecific antibody, *HER2* Human epidermal growth factor receptor 2, *IDO1* Indoleamine 2,3-dioxygenase 1, *NSCLC* Non-small cell lung cancer, *HNSCC* Head and neck squamous cell carcinoma, *HNC* Head and neck cancer, *HCC* Hepatocellular Carcinoma, *GEJC* Gastroesophageal junction cancer, *MCC* Merkel cell carcinoma, *AJCC* American Joint Committee on Cancer

## Conclusions and perspective

The response rate to monotherapy with PD-1/PD-L1 inhibitor is relatively low; thereby, optimizing combination strategies holds promise for significantly enhancing clinical efficacy of ICIs. The combination of PD-1 inhibitors with LAG-3 or CTLA-4 inhibitors has received FDA approval for use in cancer treatment. TIM-3 is an emerging and promising therapeutic target for ICIs. TIM-3 interacts with its ligands to mediate immune suppression. TIM-3 is predominantly expressed in terminally exhausted T cells and often co-expressed with PD-1. The PD-1 inhibitor upregulates TIM-3, thereby facilitating acquired resistance to anti-PD-1 treatment. TIM-3 inhibitors hold the potential to address resistance to anti-PD-1/PD-L1 therapies.

Preclinical and clinical studies across various cancer types have validated that combined inhibition of TIM-3 and PD-1/PD-L1 yields synergistic effects, proving to be more effective in tumor prevention than single-target interventions. The expression levels of TIM-3 and PD-1/PD-L1 within TME significantly influence the efficacy of PD-1/PD-L1 blockade. Mechanically, the simultaneous inhibition of TIM-3 and PD-1 can synergistically exert anti-tumor effects by regulating CTLs and enhancing multiple processes within the cancer-immune cycle. Numerous clinical trials are currently conducted to comprehensively evaluate the efficacy of this combination with anti-PD1/PD-L1, anti-TIM-3, and anti-PD-1/TIM-3 BsAg. Moreover, combining TIM-3, PD-1, and LAG-3 (or CTLA4) inhibitors are explored in clinic trails to improve immunotherapy efficacy.

Furthermore, various personalized cancer immunotherapies, such as Chimeric Antigen Receptor T (CAR)-T cell therapy, adoptive cell transfer, and therapeutic vaccines, have garnered increasing interest in recent years. Inappropriate usage of combination medications can result in greater toxicities and higher medical costs for patients. The challenges in the development of combination therapies may lie in identifying appropriate combination treatments, the lack of biomarkers that predict treatment responses, and the absence of standardized methods for measuring these parameters. Another major problem is optimizing the administration regimen, which includes dosage, timing, and sequencing.

In summary, the concurrent inhibition of TIM-3 and PD-1/PD-L1 pathway holds considerable potential in enhancing the efficacy of cancer immunotherapy. The primary aim of the preclinical and clinical research associated with this combination is to identify predictive biomarkers and develop appropriately personalized treatment strategies to augment anti-tumor immune responses, ultimately striving for the eradication of cancer cells.

## Data Availability

All data and material are available in the main text.
